# 2024 Acknowledgment of JB *Ad Hoc* Reviewers

**DOI:** 10.1128/jb.00017-25

**Published:** 2025-02-20

**Authors:** George A. O’Toole

**Affiliations:** 1 Department of Microbiology and Immunology, Geisel School of Medicine at Dartmouth 12285 , Hanover, New Hampshire, USA

## EDITORIAL

Every year, *ad hoc* reviewers make critical contributions to the *Journal of Bacteriology* (JB) by reviewing papers in their field of expertise. This year, we acknowledge 328 such reviewers who reviewed at least once between 1 January and 31 December 2024. These *ad hoc* reviewers include senior researchers who are former members of the editorial board and still do a great deal of service for the journal, as well as investigators early in their careers, including our early career researchers (ECRs), who will be the editorial board members and editors of the future. I would like to thank you all! There are many journals and many requests to review—I wanted to say a personal “thank you” to all of those who dedicated their time to review for JB.

Kaleb Abram

Fernanda Abreu

Jeella Z. Acedo

Shin-Ichi Aizawa

Ilyas Alav

Stéphane Alexandre

Salvador Almagro-Moreno

Aathmaja Anandhi Rangarajan

Christopher J. Anderson

Jeffrey C. Anderson

Mark T. Anderson

Haike Antelmann

Sandra K. Armstrong

Rachel Austin

Swapan K. Banerjee

Rui Bao

Sonia L. Bardy

Joyoti Basu

Partha Basu

Pascale B. Beauregard

Kelly S. Bender

Ralph Bertram

Tanmay A. M. Bharat

Souvik Bhattacharyya

James E. Bina

Wulf Blankenfeldt

Melanie Blokesch

Robert M. Blumenthal

Elizabeth Boon

Bradley R. Borlee

Dustin Bosch

Jeffrey S. Bourgeois

Barbara M. Bröker

L. Jeannine Brady

Konstantin Brodolin

Isabelle Broutin

Daniel R. Brown

Eric D. Brown

Pamela J. B. Brown

Sam Brown

Paula Bustamante

Bettina A. Buttaro

Matthew T. Cabeen

Wentong Cai

Elizabeth Campbell,

Bin Cao

Valerie Jean Carabetta

Jeffrey Carey ECR

Jason A. Carlyon

Ronan K. Carroll

Clayton C. Caswell

Yunrong Chai

Josephine R. Chandler

Joshua Chandler

Alain Charbit

Xavier Charpentier

Alice Chateau

Som S. Chatterjee

Francisco P. Chavez

Ambrose L. Cheung

Kotaro Chihara

Peter Chivers

Shan-Ho Chou

Fabian Commichau

Fabian Moritz Commichau

Gregory M. Cook

Brian Crane

Carole Creuzenet

Jan-Ulrik Dahl

Yunfei Dai

Ajai A. Dandekar

Richard A. Daniel

Mark R. Davies

Luiz Pedro Sorio de Carvalho

Ronnie de Jonge

Dalia Denapaite

Damien P. Devos

Nicholas A. Dillon

Li Ding

Gillian R. Douce

Djamel Drider

Richard Ducatelle

Edward G. Dudley

Maria Dulay

Michelle Dziejman

Adrianne N. Edwards

Edward H. Egelman

Jerry Eichler

Bart A. Eijkelkamp

Thomas Eitinger

Courtney K. Ellison

Gilbert Eriani

Jorge C. Escalante-Semerena

Prahathees J. Eswara

Benjamin Ezraty

Pal Falnes

Huizhou Fan

Sébastien P. Faucher

Paul D. Fey

Kenneth A. Fields

Derek J. Fisher,

Matthew Flick

Ana L. Flores-Mireles (ECR)

Kristi L. Frank

Elaine R. Frawley

Anne Galinier

Maria-Trinidad Gallegos

Erin C. Garcia

Ravindra Garde

Barney Geddes

Christopher Geiger

Abhrajyoti Ghosh

Frank C. Gibson

Steven R. Gill

Zemer Gitai

Laura A. Gonyar

Lawrence Goodridge

Revathi Govind

Marcin Grabowicz

Joerg Graf

Jeffrey A. Gralnick

Todd A. Gray

Elisabeth Grohmann

Jingmin Gu

Mingzhe Guo

Nina Molin Høyland-Kroghsbo

Pryce L. Haddix

Sven Hammerschmidt

Joe Harrison

Elizabeth L. Hartland

Eric T. Harvill

Georg Hausner

Christopher Hayes

Lizbeth Hedstrom

David E. Heinrichs

Christophe Herman

Penelope Higgs

Russ Hille

Ethan Hillman

Alexander R. Horswill

Joseph Horzempa

Andrew J. Hryckowian

Ye Huang

Ryan C. Hunter

TuAnh N. Huynh

Geelsu Hwang

Artem Isaev

Salim T. Islam

Shariful Islam

Fabrice Jean-Pierre (ECR)

Yong-Su Jin

Kathryn M. Jones

Matthew A. Jorgenson

Peter Jorth

Nathalie Juge

Sheryl Justice

Amruta A. Karbelkar

Hwan Keun Kim

Maia Kivisaar

Eric A. Klein

Leigh Knodler

Hans-Georg Koch

Matthias Koch (ECR)

Seiji Kojima

Roberto Kolter

Jens Kreth

Christopher J. Kristich

Anna Kuchina

Andrei Kuzminov

Frank E. Löffler

Antonio Lagares

Olga Maria Lage

Gyanu Lamichhane

Cristina Landeta

Robert Landick

Ruby Law

Frederique Le Roux

Michele LeRoux (ECR)

Bruno P. Lima

Trevor Lithgow

J.-M. A. Lobaccaro

Zach Lonergan

Stephen Lory

Gabriel L. Lozano (ECR)

Xuesong Luo

Amy T. Ma

Subramony Mahadevan

Grazyna Majkowska-Skrobek

Santi M. Mandal

Mark J. Mandel

Marilis V. Marques

Willm Martens-Habbena

Juan F.Martin

Melissa J. Martin

Bruce A. McClane

Patrick Julian Mester

Justin R. Meyer

Scott A. Minnich

Angela Marie Mitchell

Tim Miyashiro

Anne Moir

Gert Nikolaas Moll

Yasu S. Morita

Galina V. Mukamolova

Sampriti Mukherjee

Biswarup Mukhopadhyay

Katsuhiko Murakami

Ranjani Murali

Michael Murphy

Dennis J. Nürnberg

Carey Nadell

Beiyan Nan

Franz Narberhaus

David E. Nelson

Dianne K. Newman

Hayley J. Newton

Thomas D. Niehaus

Dietrich H. Nies

Guoqing Niu

Evgeny Nudler

Michael O'Connor

Expery Omollo

Benjamin Joseph Orlando

John Orlowski

Mehmet A. Orman

Ana P. Tedim

Jon E. Paczkowski

Yi-Jiun Pan

Matthew Parsek

Alexander B. Pastora

Shelley M. Payne

Zhong Peng

Alexandre Persat

Everett C. Pesci

Brad Pierce

Michael B. Prentice

Christopher L. Pritchett

Richard A. Proctor

Stefan U. Pukatzki

Georgiana E. Purdy

Ute Römling

Uttam L. Rajbhandary

C. S. Raman

Giordano Rampioni

Amelia M. Randich

Courtney Reichhardt

Xiaomei Ren

Nigel Robinson

Daniel D. Rockey

Kyle H. Rohde

Benjamin Ross

Dean A. Rowe-Magnus

Daniel Rozen

Jeanne Salje

Scott Samuels

Laura M. Sanchez

Jochen Schmid

Lutz Schmitt

Dirk Schnappinger

Jessica A. Scoffield

Patrick R. Secor

Gil Segal

H. Steven Seifert

Pascale Servant

Zongze Shao

Gauri Shetye

Kaixiang Shi

Robert Shields

Simon D. Silver

Gloria Soberón-Chávez

Jörg Soppa

Joseph A. Sorg

John R. Spear

Emily St. John

Christina L. Stallings

Michael N. Starnbach

Benjamin J. Stein

Madison E. Stellfox

Ann M. Stevens

David Cole Stevens

Randy B. Stockbridge

Roman Stocker

Gurol Suel

Matthew J. Sullivan

Xingmin Sun

Yi-Cheng Sun

W. Edward Swords

Constantin Nicolae Takacs

Ming Tan

Martin Thanbichler

Jenny-Lee Thomassin

Anna D. Tischler

M. Stephen Trent

Boo Shan Tseng

Paul E. Turner

Raymond J. Turner

Anne-Catrin Uhlemann

Christopher van der Gast

Vittorio Venturi

Amy C. Vollmer

Jay Vornhagen (ECR)

Samuel Wagner

Kevin J. Waldron,

Kevin John Waldron

Xindan Wang

Matthew J. Wargo

Martin J. Warren

Sivaramesh Wigneshweraraj

Brian J. Wilkinson

Julia Willett (ECR)

Leslie A. Wolf

Jerry Woo

Guoqing Xia

David C. H. Yang

Zhaomin Yang

Jinki Yeom

Alejandra Yep

Ryland F. Young,

David Zamorano-Sánchez

Raz Zarivach

Michael Zasloff

Lanying Zeng

Helen I. Zgurskaya

Changyi Zhang

Jue Zhang

Guangming Zhong

Wenhan Zhu

Wolfram Zückert

